# Using Photovoice to Examine Physical Activity in the Urban Context and Generate Policy Recommendations: The Heart Healthy Hoods Study

**DOI:** 10.3390/ijerph16050749

**Published:** 2019-03-01

**Authors:** Pedro Gullón, Julia Díez, Paloma Conde, Carmen Ramos, Valentín Márquez, Hannah Badland, Francisco Escobar, Manuel Franco

**Affiliations:** 1 Social and Cardiovascular Epidemiology Research Group, School of Medicine, Universidad de Alcala, Alcala de Henares, 28871 Madrid, Spain; pedro.gullon@edu.uah.es (P.G.); julia.diez@uah.es (J.D.); p.conde@uah.es (P.C.); francisco.escobar@uah.es (F.E.); 2 Urban Health Collaborative, Drexel Dornsife School of Public Health, Philadelphia, PA 19104, USA; 3 Public Health Institute of Madrid, Madrid City Council, 28007 Madrid, Spain; ramosmc@madrid.es; 4 Social Services Department of Madrid, Madrid City Council, 28007 Madrid, Spain; vmarquez@ucm.es; 5 Department of Social Work and Social Services, Universidad Complutense de Madrid, 28040 Madrid, Spain; 6 Center for Urban Research, RMIT University, Melbourne, 3000, VIC, Australia; hannah.badland@rmit.edu.au; 7 Department of Geology, Geography and Environmental Sciences, University of Alcalá, 28801 Alcalá de Henares, Madrid, Spain; 8 Department of Epidemiology, Johns Hopkins Bloomberg School of Public Health, Baltimore, MD 21205, USA

**Keywords:** physical activity environment, urban health, participatory action research, photovoice

## Abstract

A current challenge in physical activity research is engaging citizens with co-creating policies that support physical activity participation. Using Photovoice, a participatory action research method, the objectives of this study were to: 1) Identify community perceptions of urban built, social, and political/economic environment factors associated with physical activity; and 2) generate community-driven policy recommendations to increase physical activity. Two districts in Madrid of varying socio-economic status (SES) were selected. Overall, 24 residents participated in 4 groups stratified by sex and district (6 participants per group). Groups met weekly for 4 weeks to discuss and analyze their photographs. Participants coded photographs into categories, which were then regrouped into broader themes. The categories were transformed into policy recommendations using an adaptation of the logical framework approach. Participants took 161 photos, which were classified into 61 categories and 14 broader themes (e.g., active transportation, sport in the city). After this, participants generated a set of 34 policy recommendations to improve the urban environment to support physical activity (e.g., to redistribute sports facilities). Collaboration between citizens and researchers led to a deeper understanding of the community perceptions of urban built, social, and political/economic environment factors associated with physical activity in two districts of Madrid, while engaging citizens in recommending public policies.

## 1. Introduction

Insufficient physical activity is one of the 10 leading risk factors for deaths worldwide, causing an estimated 3.2 million deaths each year [1]. In fact, lack of sufficient physical activity has been estimated to cause a 2.8% loss of total global annual Disability-Adjusted Life Years (DALYs) [1]. In particular, physical inactivity has a population attributable factor of 5.8% for coronary heart disease, 7.2% for type 2 diabetes, 10.1% for breast cancer, 10.4% for colon cancer, and 9.4% for the overall mortality [2]. Despite this, physical inactivity levels in European countries remain high, especially in Ireland, Italy, Malta, Portugal, and Spain [3]. For instance, using data from the European Social Survey, Marques et al. estimated that 39% of European adults (>18 years old) did not reach the World Health Organization (WHO) physical activity recommendations [4]. Moreover, physical activity follows a social gradient, where the most disadvantaged populations are less likely to meet physical activity recommendations [5,6].

Cities and neighborhoods provide many opportunities to support physical activity through the built, social, and political environments [7]. For example, many characteristics of the urban environment, such as neighborhood walkability or the availability of parks, have been studied as determinants for physical activity in different settings [8,9,10]. One of the challenges arising from this research is understanding the role of planning and policy, alongside how best to engage citizens in forming recommendations for policy change.

Participatory action research (PAR) is a suitable approach for involving citizens in research and policy change recommendations. Specifically, community-based participatory research (CBPR) is an approach that acknowledges the community as an equal partner throughout the research and action processes [11]. CBPR methods include actively engaging communities in the research processes to promote changes [12]. Photovoice is a participatory method coming out of CBPR and PAR paradigms and is defined as “a process by which people can identify, represent, and enhance their community through a specific photographic technique” [13]. Photovoice has been used previously as a method for identifying key elements of the neighborhood that are important for physical activity in adults [14,15,16] and in children [17,18]; however, the potential of Photovoice methodology for translating residents’ voices into policy change for active living is still in its first steps [19,20,21]. Also, it has been shown to be more useful than qualitative methods alone in understanding the urban context, as it incorporates discussions from specific neighborhood elements through photography [22]. Photovoice can also be included under the umbrella of “citizen science” methods, where residents document their physical and social environment and their findings are used to advocate for change [23].

The objective of this study was two-fold, being to: (1) Identify, using Photovoice, community perceptions of urban factors associated with physical activity in two different socioeconomic (SES) neighborhoods in Madrid; and (2) generate community-driven policy recommendations to increase physical activity in the urban environment.

## 2. Materials and Methods

### 2.1. Project Design

This research was conducted in two phases. First, we conducted five Photovoice group sessions to identify aspects of the urban environment that residents considered as relevant for physical activity (phase 1). Second, in two additional sessions, participants generated a set of policy recommendations to improve physical activity levels in their neighborhoods (phase 2).

This Photovoice project took place between October 2016 and June 2017 in Madrid, as part of the Heart Healthy Hoods study (hhhproject.eu), which studies the relationship between the physical and social urban environment and the cardiovascular health of residents living in Madrid, Spain [24,25]. We conducted this study in accordance with the Declaration of Helsinki, and received ethical approval by the Ethics Committee of the Universidad de Alcala (CEI/HU/2017/09).

### 2.2. Setting

We purposively selected two different administrative districts of Madrid (named Villaverde and Chamberí) to capture variability in the socioeconomic (SES) and spatial distribution of the city. Chamberí is a high-SES district close to the city center, whereas Villaverde is a low-income district in the periphery of the southern part of Madrid. A summary of the SES and demographic characteristics of both areas can be found in Table S1 (Supplementary Material) [26].

### 2.3. Participants and Sample

To engage Photovoice participants, we based their recruitment on the residence location and used a purposive sampling strategy [27]. We included adult men and women that: (1) Have lived in their respective neighborhoods for more than one year; (2) spoke Spanish; (3) had no impediments to manage a digital camera; and (4) agreed to attend the group discussion sessions of the two phases.

With the objective of capturing different “voices”, we tried different recruitment strategies. For example, we distributed information sheets and flyers and organized community meetings with neighborhood associations. The project and the eligibility requirements were explained to a contact person at each neighborhood association who assisted with recruitment. The local Community Health Center in Villaverde and the Social Services Department in Chamberí facilitated the recruitment and retention. There was no economic incentive to participate in the study; however, participants received a personal portrait taken by the professional photographer as an acknowledgment of their participation. Participants were fully informed about the project and signed the consent form.

### 2.4. Project Phases

#### 2.4.1. Phase 1: Photovoice Process about Urban Environmental Factors Associated with Physical Activity

Four Photovoice groups were set up, two in Chamberí and two in Villaverde; stratified by neighborhood and sex: (a) Six women in Chamberí, (b) 6 men in Chamberí, (c) 6 women in Villaverde, (d) 6 men in Villaverde. Wang has suggested 7 to 10 participants as the ideal group size for Photovoice [28]; however, smaller groups have been found to be effective for the purposes of the Photovoice methodology [20].

Groups met weekly for five sessions that each lasted ~2 h. The women group in Villaverde had an additional session to complete the tasks of the last session. Each session was facilitated, at least, by an academic-based researcher and a facilitator from the partner organizations (Social Services Department at Chamberí and Health Promotion Center at Villaverde). All sessions were recorded and transcribed.

In the first session, a brief introduction to the project and on the content of each of the five sessions was provided. In this session, participants filled out the informed consents and a demographic questionnaire. A professional photographer assisted with this session by offering training in photography. The one-hour training session covered personal safety, ethics, and responsibilities of being the photographer, and instructions on the photography assignments for the project. Participants received a digital camera, which was returned at the end of the five sessions. At the end of this first session, participants were asked to photograph features of their respective neighborhoods that they perceived as facilitators or barriers for physical activity. We defined physical activity as “any physical movement or mobility carried out for the purpose of leisure (e.g., walk in the park, dance class, workout at the gym) or transportation (e.g., walking/cycling to a destination)” [16]. We asked participants to take as many photos as relevant; however, only a maximum of five photos per participant could be brought to the next session to better focus the debate [29].

For session 2, each participant arrived with up to five photos. We used a modified version of the SHOWED mnemonic method to guide the discussions of the photographs [13]; our adaptation included the following questions: (1) What do you see in the photo? (2) What is the story behind the photo? (3) How does this relate to physical activity? We decided to make these changes after a previous experience in another Photovoice project in Madrid [20], where some of the SHOWED questions were not completely understood by participants. These revised questions allowed a dialogue process that draws on Paulo Freire’s notions of empowerment education [30] to bring the perspectives and voices of community members into the research process. Facilitators only intervened to clarify doubts or to involve participants that were not participating actively in the discussions. In sessions 3 and 4, residents could either bring new photos or continue with the discussion from the previous sessions. In session 5, participants classified their photos into categories and decided which photographs were going to be selected for dissemination purposes.

#### 2.4.2. Phase 2: Development of Community-driven Policy Recommendations to Increase Physical Activity in the Urban Environment

In this phase, we merged men and women groups in each of the settings. Thereby, we had one group in Chamberí (*N* = 12) and one group in Villaverde (*N* = 12). First, participants presented their results to the wider group. Following on, participants identified policy recommendations using an adapted logical framework approach [31]. The logical framework approach is usually used to identify community necessities, to describe their problems and their desired improvements as well as the possible solutions [21].

From the participants’ main results (phase 1), they built a problems tree. This problems tree consisted of a trunk, which represented the main problem (e.g., unhealthy physical activity environment); from this trunk, several tree knots represented specific problems (e.g., difficulty to use bikes); from the tree knots, hanging roots represented the causes (e.g., insufficient bike lanes). Once the tree was built, participants reformulated it into a solutions tree. For example, the specific problems were converted into solutions (e.g., improve the use of bikes) and the roots were converted into policy recommendations (e.g., increase the number of bike lanes).

### 2.5. Data Analysis

Participants discussed their photos and coded the issues, themes, or topics that arose from their photos [13]. Therefore, in the last group session of phase 1, we asked the group to reflect (code) on the patterns or categories that had emerged in the photos and stories/narratives. Accordingly, participants classified their photos into categories and then they selected one representative photo of this category. They also chose the final photographs they wanted to be included in the dissemination activities (e.g., photobook or the photography exhibition). We then grouped these categories into broader themes using the deductive analytical strategy of ‘successive approximation’, a method ‘of qualitative data analysis in which the researcher repeatedly moves back and forth between the empirical data and abstract concepts or theories’ [16,32]. Then, we cross-checked the broader themes with participants for their agreement.

For the analysis of our second objective, we used the Analysis Grid for Environments Linked to Obesity (ANGELO) framework, and we classified their policy recommendations into: (1) Physical environment recommendations, (2) sociocultural environment interventions, or (3) political and economic interventions [14,33].

## 3. Results

Table 1 presents the main sociodemographic characteristics of the 24 Photovoice participants. Participants’ ages ranged from 34 to 72 years (median = 55). Two participants were foreign-born and lived in Villaverde. In Villaverde, nine participants lived with monthly household incomes lower than 1200 €; within these, three had monthly household incomes lower than 600 €. In Chamberí, all participants had at least a college degree and had monthly household incomes higher than 1200 €.

Participants took a total of 161 photos related to the physical activity environment in Villaverde and Chamberí. From these photographs and their discussions, they identified 35 categories in Villaverde and 26 in Chamberí. These categories were then grouped into 14 broader themes, as shown in Table 2. Results for phase 1 are presented according to the 14 broader themes, using participants’ selected photographs, their SHOWED-based narratives, and other group members’ related discussions. First, the common categories in both districts of Villaverde and Chamberí are presented, and then the specific categories for each area. For phase 2, participants’ policy recommendations are presented.

### 3.1. Phase 1: Photovoice Results about Urban Environmental Factors Associated with Physical Activity

#### 3.1.1. Common Themes Highlighted in Both Districts (Figure 1)

**Figure 1:**
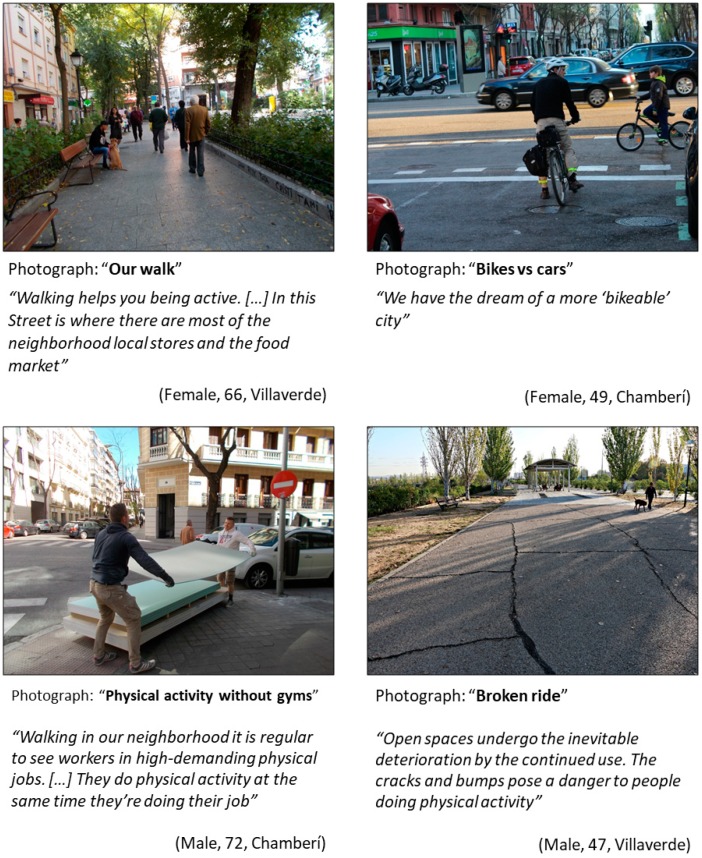
Photos representing common themes in Villaverde and in Chamberí. (Upper-left) Photograph: “Our walk”; category: Pedestrian spaces; theme: Active transportation. (Upper-right) Photograph: “Bikes vs cars”; category: Individual effort to change mobility behavior; theme: Active transportation. (Bottom-left) Photograph: “Physical activity without gyms”; category: Working as physical activity; theme: Working as physical activity. (Bottom-right) Photograph: “Broken ride”; category: Maintenance of public spaces; theme: Local administrations.

1. Active transportation. The theme of active transportation arose in the four Photovoice groups. Participants reported that the role of active transportation (e.g., walking or biking for daily activities) was one of their main modes of physical activity (Figure 1 upper-left and upper-right photographs); for instance, one 47-year-old man from Villaverde reported: “*urban environments happens to be used for walking and citizen meeting. It is a place to share and integrate, that is why we must keep it in optimal conditions*”. Residents also discussed the changes in the perception of active transportation; regarding this issue, one 65-year-old man of Chamberí said: *“We are living the integration of physical activity in daily activities and the breaking of the traditional norms of displacement in the city, as has already happened in many other European capitals”.*
2. Working as physical activity. Participants reported that some jobs demanded higher levels of physical activity. Discussing the bottom-left photo in Figure 1, a 72-year-old man of Chamberí said that “*this exercise is paid with a salary and is developed every day in equivalent times (in hours) to those dedicated by elite athletes for their training*”. They also discussed the growth of foot or bicycle delivery companies in Madrid (e.g., Deliveroo^®^ (London, United Kingdom), JustEat^®^ (London, United Kingdom)) and how these demanding jobs did not leave energy for the rest of the daily activities; for instance, a 44-year-old woman from Villaverde claimed “*working while doing exercise... I would feel exhausted!*”
3. Local administrations. The role of the local administrations, such as the city council or the regional government, was a core theme across the four groups. As shown in Figure 1, bottom-right panel, participants highlighted that there were ‘abandoned’ areas in their neighborhood, and this could be problematic for physical activity. On the other hand, Chamberí participants discussed the availability of public sports facilities and the privatization of these facilities in the last years. For instance, a 40-year-old woman said: “*The only municipal sports center in the neighborhood is an adjudication to a private company. The neighbors do not have priority, and although there is public control of prices, it is too expensive to become a member. If you are not a member, it is impossible to access the activities that are offered, it is useless, then people look for leisure alternatives, being healthy or not*”.

#### 3.1.2. Specific Themes from Villaverde District (Figure 2)

**Figure 2:**
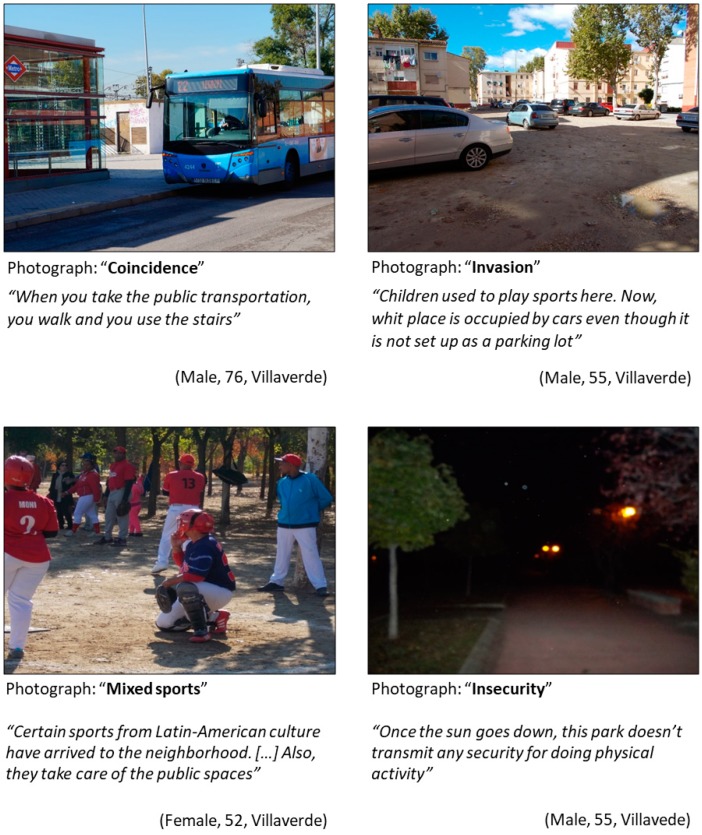
Photos representing specific themes in Villaverde. (Upper-left) Photograph: “Coincidence”; category: Lack of communication between different parts of the neighborhood; theme: Public transportation. (Upper-right) Photograph: “Invasion”; category: Reuse of public spaces; theme: Public spaces. (Bottom-left) Photograph: “Mixed sports”; category: Mixed sports; theme: Citizens’ awareness. (Bottom-right) Photograph: “Insecurity”; category: Safety; theme: Safety.

1. Public transportation. As shown in the upper-left panel in Figure 2, participants from Villaverde recognized using public transportation was a way to incorporate physical activity in daily life. Participants stressed the importance of the availability of the public transportation infrastructure. On the one hand, they highlighted that Villaverde was well-connected with the rest of the city. On the other hand, they also identified the lack of communication within different parts of the neighborhood, as a 55-year-old participant argued: “*the only bus line that connects the neighborhood doesn’t have service on Sundays and holidays. They do not give service to other areas of the neighborhood and especially the sports center*”.
2. Public spaces. Residents emphasized that public open spaces were important places for physical activity. They also discussed the quality of the public spaces, and how cars have changed the use of public spaces that were previously used for physical activity, as is depicted in the upper-right photograph and text of Figure 2.
3. Citizens’ awareness. One of the main topics discussed in the Photovoice sessions in Villaverde was the importance of citizens’ awareness. Participants argued that recognition of diversity (e.g., gender, ethnicity) was an important consideration for physical activity promotion (bottom-left panel Figure 2). They also highlighted that there was a need for education of the public to take care of public spaces, as a 76-year-old man noted: “*Education is an important factor for the good maintenance of the means that the public administration provides, without the zeal and the care of these means doing physical activity would not be possible*”.
4. Safety. Safety was considered as one important limitation for physical activity, as it can be seen in the photograph and verbatim in the bottom-right panel of Figure 2. Residents were worried that, despite the availability of public spaces, they could not use them for safety issues. For instance, a 47-year-old man discussed, when talking about a public square “*The degradation of the plaza and the lack of security make it an unsafe place for adults and children*”.

#### 3.1.3. Specific Themes from Chamberí District (Figure 3)

**Figure 3:**
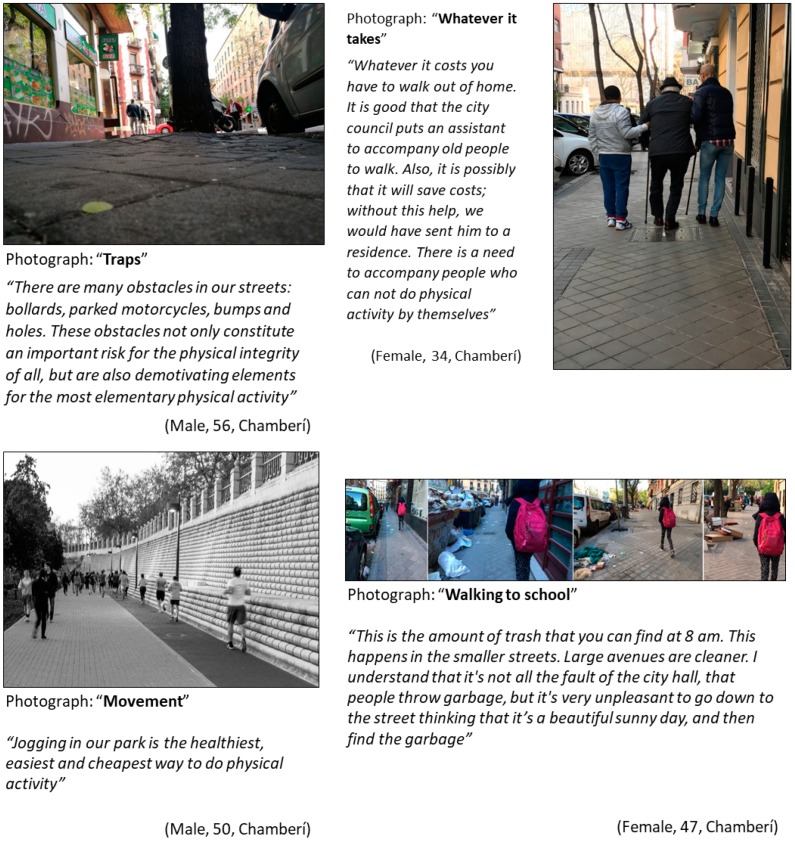
Photographs representing specific themes in Chamberí. (Upper-left) Photograph: “Traps”; category: Streets and sidewalks; theme: Urban architecture. (Upper-right) Photograph: “Whatever it takes”; category: Need to support the elderly; theme: Physical activity for all social groups. (Bottom-left) Photograph: “Movement”; category: Physical activity at ‘Canal park’; theme: Sport in the city. (Bottom-right) Photograph: “Walking to school”; category: Antisocial behavior; theme: Antisocial behavior.

1. Urban architecture. Participants understood the urban context as the urban infrastructure, streets, and pavement. Overall, participants considered Chamberí as a walkable neighborhood, where they could do most of their daily activities within a walking distance. Despite this, they identified a need for improvement in the maintenance of streets, especially for vulnerable people, as shown in the upper-left panel in Figure 3.
2. Physical activity for all social groups. In this theme, participants discussed active aging, physical activity adapted to all the family members, and the need to support old people (Figure 3 upper-right panel). They also discussed the positive effects of doing physical activity in groups, despite the time and economic resources needed to participate. A 72-year-old participant declared: “*Jogging in the running circuit is aimed at a privileged sector of people among those who do physical activity in Chamberí. This group has an exercise plan, spends a time on that plan, pays club fees and they surely return to the gym running*”.
3. Sport in the city. Participants identified numerous places where to practice sport in their neighborhoods, such as open spaces (parks, squares, streets) and roofed spaces (gyms, sports clubs). In particular, they placed an emphasis on the sports activities that took place in the “Canal Park”, the largest in the neighborhood, and with different sports equipment (Figure 3 bottom-left panel).
4. Antisocial behavior. Participants identified antisocial behavior in the neighborhood that discouraged physical activity. As seen in Figure 3 bottom-right panel, participants discussed the presence of litter and trash in the public spaces and streets. They also pointed out that other vehicles occupy spaces reserved for pedestrians, such as sidewalks. For example, a 64-year-old woman said: “*Motorbikes have become a well-established mean of transportation in Chamberí. The problem is that there are not enough car lots and they are invading the sidewalks*”.

### 3.2. Phase 2: Development of Community-Driven Policy Recommendations to Increase Physical Activity in the Urban Environment

Following the logical framework approach, participants identified 34 policy recommendations related to physical activity in urban settings (19 = Villaverde, 15 = Chamberí). Table 3 provides an overview of the entire set of physical, sociocultural, economic, and political environmental policy recommendations made by the participants.

Regarding the physical environment, participants identified six policy recommendations in Villaverde, and five in Chamberí. Participants recommended greater access to resources (e.g., to redistribute sports facilities) and improvements in the maintenance of streets and public spaces (e.g., sidewalk maintenance). Regarding the sociocultural environment, residents identified eight recommendations (four in each district). They advocated for education campaigns on physical activity (e.g., to educate in the practice of mixed-gender physical activity or to design active transportation awareness campaigns). Finally, a set of 16 recommendations (9 = Villaverde, 7 = Chamberí) related to the political (e.g., to limit traffic speed to increase pedestrian safety) and economic (e.g., to adjust sport facilities fees to the area-level socioeconomic status) environment.

## 4. Discussion

Through this Photovoice project, residents from two socioeconomically different neighborhoods in Madrid engaged as co-researchers in a participatory process where they discussed their local environment in terms of physical activity, analyzed the data, and generated a set of policy recommendations to support physical activity.

In Phase 1, participants from Villaverde and Chamberí identified three common and eight neighborhood-specific themes that were related to their physical activity environment and the practice of physical activity. The common themes were: (1) Active transportation, (2) working as physical activity, and (3) the local administrations. Active transportation, such as walking or cycling for daily life activities, has been identified as an independent predictor of overall physical activity and better cardiovascular health in previous quantitative studies [34]. However, residents added new evidence on how the perception and acceptance of different modes of transportation are changing in Madrid, one of the most polluted cities in Europe [35]. Residents also added new insights into work-related physical activity, especially on how new jobs in the collaborative economy field [36] can be both an ‘opportunity’ for work-related physical activity as well as a risk for a precarious job and lack of time in other daily life activities. The role of the public administration in co-creating healthier environments with citizens and researchers is gaining traction in neighborhoods and health fields [37]. In the present study, residents from Villaverde discussed how they felt ‘abandoned’ by the public administrations, specifically in the management of public spaces.

Residents also identified specific categories for their neighborhoods. These characteristics, such as public spaces, safety, public transportation, or the role of urban architecture, have been identified previously as key environmental elements for the promotion of physical activity [8,38,39]. For instance, safety was identified as an emerging theme in Villaverde (low-SES neighborhood), which aligns with other Photovoice projects where residents of low-SES communities in San Diego identified safety as a barrier for physical activity [40]. We found some differences in the themes and discourses between both settings. For instance, the themes, “citizens’ awareness”, (Villaverde) and “antisocial Behavior” (Chamberí), may look similar, but the connotations are quite different. While in Villaverde, the issue of “citizens” awareness” had an “our community has to do” approach, in Chamberí, the “antisocial behavior” was seen as something external, “others have to do”.

Our study provides additional evidence to the existing literature. For example, residents from Chamberí identified the lack of physical activity offered for different social groups as a barrier to the population’s physical activity. Moreover, residents talked about specific issues that would not arise in regular quantitative methods. For instance, participants from Villaverde expressed that their availability of public transportation was a facilitator for transport-related physical activity to the rest of the city of Madrid; however, they also identified the lack of connectivity by bus between different parts of the neighborhood as a barrier to walking in the neighborhood. Although participants in this study acknowledged the importance of ecological factors in cardiovascular health, they also emphasized personal responsibility, such as the importance of ‘citizens’ awareness’ in Villaverde or ‘antisocial behavior’ in Chamberí. Public health and urban health discourse have emphasized in the last years the importance of ecological factors on health conditions; however, as evidenced by this study and others [41], individuals might be accepting of other individualistic and personal responsibility perspectives due to the individualistic ideology hegemonic in modern society [42].

Residents’ photographs, discussions, and categories served as the starting point for developing the 34 recommendations in phase 2 (shown in Table 3). Within these recommendations, some were related to the physical (e.g., to create new bike lanes), the sociocultural (e.g., to educate in the practice of mixed-gender physical activity), and the political environment (e.g., to maintain public management in public spaces and facilities). The participation of citizens in the design of healthy urban policies is a key element for their success [43]. On the one hand, Villaverde residents identified the need for a re-designing of the neighborhood’s internal transportation network to encourage active transportation. On the other hand, residents of Chamberí identified that, despite having one of the largest sports facilities in Madrid, the center was used by people from other neighborhoods, and not by local residents. This information would be missing without the participatory approach adopted in this study.

The active engagement of community members in the research process demonstrates that participants are an essential part of the research team, and might also further contribute to a sense of ‘community ownership’ that will help further discussions on important neighborhood issues [13]. There is also significant value in the Photovoice process for the partner communities. The co-production of knowledge by the participants and researchers increases the impact of findings for the community. Indeed, the community partnership (in our case, with the local Health Promotion Center in Villaverde and the Social Services Department in Chamberí) could lead to unexpected changes in the future due to the ‘spillover effect’; that means that the new collaboration could work together in new aspects that were not in the original design.

As possible implications and directions for further research, we would like to highlight that participatory processes might imply important benefits for public health. As suggested by Verlinghieri [43], participatory processes can be beneficial for health in three aspects, these being to: (1) Design tailored interventions and value alternative resources for health; (2) generate empowerment-related community wellbeing as shown in our previous Photovoice project [44]; and (3) redefine authoritative new knowledge on the base of co-production. The next step of this project is to continue citizen science meetings in the community, which we have started already in Villaverde. In these meetings, participants inform policy-makers, media, and other neighbors on their main findings and their policy recommendations. With these meetings, we also open public exhibitions where the selected photographs of the participants are displayed. Another current step is the integration of all these measures into a Geographic Information System (GIS) application. With this application, we would like to disseminate the photographs and discourses into a spatial data platform, where we can also compare it with quantitative measures of the neighborhood [45].

This study presents some limitations. Firstly, we conducted this research in two neighborhoods where there is a long history of local participation and activism. However, involving residents in community processes is difficult without a history of community participation, and the selection made our project more feasible. Second, our sample size in the groups was smaller than previous studies have reported [46]; however, as pointed out in the methods section, smaller groups are also effective for reaching Photovoice objectives [20]. Third, we missed children’s “voices” in this Photovoice study, as we focused on the adult population. Fourth, we did not include policy-makers from the beginning of the process. By doing so, it could be a mechanism that improves the uptake of the policy recommendations [47]. Nevertheless, we followed a bottom-up approach, as we wanted policy-makers to be removed from the participants’ preliminary discourses and their generation of policy recommendations. One step further for future projects is to include citizen control in the follow-up of the policy implementation as the highest degree of citizen power [43]. Last, participants could only select five of their photographs to be discussed in the Photovoice groups; however, they chose the photos that they wanted to share, which followed Caroline Wang and Nykiforuk’s suggested guidelines [28,29].

## 5. Conclusions

This project created an environment in which participants from two different SES neighborhoods in Madrid discussed and reflected on the elements of the urban environment that either favored or prevented them and their peers from being physically active. Through their photographs and collective discussions, participants identified emerging categories, such as the role of public administrations, urban attributes, or citizens’ awareness. Moreover, participants generated and presented to policy-makers a set of recommendations to improve their physical activity environment in relation to the physical environment, the socio-cultural environment, and the political/economic environment. This participatory process implies a change of the perspective in the relationship between researchers, citizens, and policy-makers. Participatory research processes, such as Photovoice, are important methods to generate culturally adapted changes to meet the needs of the community, in this case, to increase physical activity levels and improve population health.

## Figures and Tables

**Table 1:** Characteristics of the participants (*N* = 24) in Villaverde and Chamberí, Madrid.

Sociodemographic Characteristics	Villaverde			Chamberí		
	Women (*N* = 6)	Men (*N* = 6)	Total (*N* = 12)	Women (*N* = 6)	Men (*N* = 6)	Total (*N* = 12)
Median age	57	51	57	47	67	53
Place of birth						
Spain	5	5	10	6	6	12
Other	1	1	2	0	0	0
Highest level of education						
College degree	3	4	7	6	6	12
High-school graduate	0	2	2	0	0	0
Not a high-school graduate	3	0	3	0	0	0
Employment						
Employed	1	4	5	5	3	8
Unemployed	2	0	2	0	0	0
Retired	1	2	3	1	3	4
Housewives	1	0	1	0	0	0
Not reported	1	0	1	0	0	0
Median household income per month						
<600 €	1	2	3	0	0	0
601–1200 €	4	2	6	0	0	0
1201–1700 €	0	2	2	2	0	2
1701–2200 €	0	0	0	2	2	4
>2200 €	0	0	0	2	3	5
Not reported	1	0	1	0	1	1

**Table 2:** Photovoice themes (*N* = 14) in Villaverde (low-SES neighborhood) and Chamberí (high-SES neighborhood) resulting from the successive approximation process.

Villaverde	Chamberí
1. Active transportation	1. Active transportation
2. Working as physical activity	2. Working as physical activity
3. Local administrations	3. Local administrations
4. Public spaces	4. Physical activity for all social groups
5. Safety	5. Sport in the city
6. Public transportation	6. Urban Architecture
7. Citizens’ awareness	7. Antisocial behavior

**Table 3:** Policy recommendations from Photovoice participants grouped according to the Analysis Grid for Environments Linked to Obesity (ANGELO) framework [14].

Domains	Villaverde	Chamberí
**Physical environment**	Redistribute sports facilities favoring proximity	Surface improvements (e.g., sidewalk maintenance)
	Re-design the bus network	Create new bike lanes
	Increase street furniture	Increase accessibility to sport facilities
	Place the existing outdoor fitness equipment in parks	Include physical activity amenities in small spaces
	Widen sidewalks for people with reduced mobility	Create pedestrian streets for walking
	Improve access to the urban gardens	
**Socio-cultural environment**	Increase awareness on civic responsibility regarding the use of public spaces	Educate in the practice of age-specific physical activity
	Delimit use of public spaces	Design active transportation awareness campaigns
	Educate in the practice of a mixed-gender physical activity	Awareness campaign against antisocial behavior
**Political and economic environment**	Adjust sport facilities fees o the area SES	Create incentives for active transportation
	Build parking lots and a bike lane	Map cultural tours for walking in the neighborhood
	Create informative signs on the use of sports facilities and public spaces	Create an app for combined transportation (walking + public transportation)
	Support residents’ initiatives and events promoting physical activity	Limit traffic speed to increase pedestrian safety
	Increase human resources at sport facilities	Limit motorbike parking in sidewalks
	Open schools’ sports facilities to community users	Open schools and other public centers for cultural and social uses
	Increase maintenance of neighborhood green spaces	Maintain public management in public spaces and facilities
	Create multipurpose spaces for diverse activities	
	Increase security in public spaces	

## Supplementary Materials

The following are available online at https://www.mdpi.com/1660-4601/16/5/749/s1, Table S1: Sociodemographic characteristics of Villaverde, Chamberí and the city of Madrid in 2017 according to the Open Data Portal of Madrid’s city council.
