# Supplementary material for: Using Photovoice to Examine Physical Activity in the Urban Context and Generate Policy Recommendations: The Heart Healthy Hoods Study
Source: Int J Environ Res Public Health. 2019 Mar 1;16(5):749. doi: 10.3390/ijerph16050749 (PMC6427388; doi:10.3390/ijerph16050749)
Supplement: Supplementary file 1 [file ijerph-16-00749-s001.pdf]

**Table S1**. Sociodemographic characteristics of Villaverde, Chamberí and the city of Madrid in 2017 according to the Open Data Portal of Madrid's city council (available at: https://datos.madrid.es/portal/site/egob/menuitem.9e1e2f6404558187cf35cf3584f1a5a0/?vgnextoid=374512b9ace9f310VgnVCM100000171f5a0aRCRD&vgnextchannel=374512b9ace9f310VgnVCM100000171f5a0aRCRD&vgnextfmt=default )

| Characteristics | Villaverde | Chamberí | Madrid city |
|---|---|---|---|
| Population density (hab/km$^2$) | 7,056.13 | 29,675.80 | 5,265.91 |
| % of > 65 years old | 18.1 | 24.2 | 20.5 |
| Median household income (€) | 24,870.78 | 49,347.6 | 40,440.1 |
| Unemployment rate | 12.1 | 6.0 | 8.7 |
| % of people 25 years old or higher with highest education secondary school or lower | 37.7 | 16.0 | 26.5 |
| % with good or very good health | 66.1 | 82.9 | 74.3 |
| % of obesity | 14.5 | 11.2 | 10.6 |
| % of sedentary population | 23.7 | 19.7 | 24.4 |
